# Secondary School Students and Caffeine: Consumption Habits, Motivations, and Experiences

**DOI:** 10.3390/nu15041011

**Published:** 2023-02-17

**Authors:** Sophie Turner, Ajmol Ali, Carol Wham, Kay Rutherfurd-Markwick

**Affiliations:** 1 School of Sport, Exercise and Nutrition, Massey University, Auckland 0632, New Zealand; 2 Centre for Metabolic Health Research, Massey University, Auckland 0632, New Zealand; 3 School of Health Sciences, Massey University Auckland 0632, New Zealand

**Keywords:** adolescents, adverse effect, caffeine, consumption patterns, energy drinks, New Zealand

## Abstract

Adolescents may be particularly vulnerable to the effects of caffeine due to a lack of tolerance, their small size, changing brain physiology, and increasing independence. Concerns about adolescent caffeine consumption relate to potentially serious physiological and psychological effects following consumption. Motivations driving caffeine intake are not well understood among adolescents but are important to understand to reduce harmful behavioural patterns. This study explored caffeine consumption habits (sources, amount, frequency) of New Zealand adolescents; and factors motivating caffeine consumption and avoidance. The previously validated caffeine consumption habits questionnaire (CaffCo) was completed by 216 participants (15–18 years), with most (94.9%) consuming at least one caffeinated product daily. Chocolate, coffee, tea, and kola drinks were the most consumed sources. The median caffeine intake was 68 mg·day^−1^. Gender (boy) and being employed influenced the source, but not the quantity of caffeine consumed. One-fifth (21.2%) of adolescents consumed more than the recommended European Food Safety Authority (EFSA) safe level (3 mg·kg^−1^·day^−1^). Taste, energy, and temperature were the main motivators for consumption, and increased energy, excitement, restlessness, and sleep disturbances were reported effects following caffeine consumption. This study provides information on caffeinated product consumption among New Zealand adolescents, some of whom consumed caffeine above the EFSA safe level. Public health initiatives directed at adolescents may be important to reduce potential caffeine-related harm.

## 1. Introduction

Globally, at least 70% of people consume caffeine, making it the most frequently and widely consumed mental stimulant. Similarly, 71–96% of New Zealanders over the age of 15 have been reported to consume caffeine. Caffeine occurs naturally in tea, coffee, and chocolate, or it may be artificially added to products such as soft drinks, energy drinks, sports gels, and medications [1]. Caffeine intake during adolescence is especially of concern as this is a developmental period during which the brain undergoes changes, including the development of the prefrontal cortex to prepare for adulthood [2]. Sleep is critically important for this brain development, but high caffeine intake is associated with sleeping problems and daytime sleepiness in adolescents [3].

Individual responses to caffeine vary according to genetic and environmental factors, as well as age, sex, and habituation to caffeine [4,5]. Adolescents are still growing, so their smaller size and lack of habituation to caffeine intake increase their risk of experiencing the negative effects of caffeine via intentional excess consumption [6]. At lower caffeine doses, cognitive effects such as improved alertness, reaction time, mood, and reduced tiredness typically occur [7,8,9]. At higher doses, there is an increased risk of experiencing the negative effects of caffeine consumption, including anxiety, jitteriness, diuresis, insomnia, and tachycardia [7,9,10,11]. As caffeine can have a diuretic effect, caution should be taken when using highly caffeinated drinks to hydrate when in a hypohydrated state [12].

Understanding how much caffeine adolescents consume and their motivation for intake are important to address due to their increased risk of exceeding a safe level of caffeine intake, which has been suggested to be between 2.5–3.0 mg·kg^−1^·day^−1^ by Health Canada and the European Food Safety Authority (EFSA) [11,13]. The reasons that adolescents use caffeine are likely to differ from that of adults or younger children [14,15], with sociocultural factors including employment status (disposable income) and cultural norms also influencing the types and amounts of caffeinated products consumed.

Previous studies have determined the amount of caffeine consumed by New Zealand adolescents, with the most recent based on data from the New Zealand Adult Nutrition Survey conducted in 2008/2009 [10]. Since that time, the availability of caffeinated products and caffeine intake patterns are likely to have changed. Therefore, the aim of the present study was to understand current caffeine use among New Zealand adolescents and understand the motivations/enablers/barriers for consumption in this age group. The caffeine consumption habits questionnaire (CaffCo) was chosen as an appropriate tool to quantitatively evaluate caffeine consumption in this age group.

## 2. Materials and Methods

### 2.1. Participant Recruitment

Secondary school students from the Auckland and Northland regions of New Zealand were invited to complete a web-based questionnaire to investigate their caffeine consumption habits between June 2016 and July 2017. Participants were recruited via social media, at university open days, shopping malls, and via teachers in a classroom setting; those aged 15 to 18 were eligible to participate. Participants who met this age requirement and completed the questionnaire were included in the study.

Caffeine consumption (or lack of it) was not an inclusion/exclusion criterion for participation. Written informed consent was gained before the commencement of the study through either paper or internet-based consent forms after potential participants were provided with an information sheet and were given an opportunity to clarify queries with a member of the research team. Those aged 15 were deemed minors and therefore required parental consent to participate.

Ethical approval for this study was granted by the Massey University Human Ethics Committee: Southern A (Application SOA 15/76).

### 2.2. Caffeine Consumption Habits Questionnaire (CaffCo)

The New Zealand-validated CaffCo was used to investigate consumption patterns [16]. The CaffCo was administered to participants via an online survey software (Qualtrics, Provo, Utah, USA). on participants’ own devices or on provided tablets (iPads, Apple Inc., Cupertino, CA, USA). Each participant was assigned a randomised six-digit identification number to ensure responses were anonymous.

The CaffCo comprises 49 sections, which examine the frequency and amount of caffeine consumed from eight sources: tea, coffee, chocolate, energy drinks, kola drinks (NB, the term kola is used to differentiate between the type of soft drink and the brand Coca-Cola), caffeinated ready-to-drink alcoholic beverages (RTDs; e.g., bourbon and kola drinks), sports supplements, and caffeine tablets.

The questionnaire collected demographic information including age, sex, ethnicity, self-reported weight (kg) and height (cm), employment status (whether they were a student, unemployed, employed part time or employed full time), the highest level of education enrolled in or completed, living situation (alone, with family, in a family situation or other) whether they smoke, and the current use of oral contraceptives (women only).

Questionnaire items prompted the respondents for their motivation to consume each caffeinated product from a list of 15 to 26 options per product (potential motivation for consumption was determined from focus groups and existing literature during the development of the CaffCo [16]. Thereafter, the reasons for not consuming caffeinated products were explored using a list of nine options per product. Finally, participants were questioned about the effects they may have experienced following caffeine intake and which products they associated with each effect.

### 2.3. Data Analysis

#### 2.3.1. Body Mass Index

For participants who self-reported their height and weight (*n*= 133), body mass index (BMI) was calculated, and respondents were categorised as thin/underweight, normal, overweight or obese using the 2007 World Health Organization BMI-for-age charts for boys and girls, respectively [17].

#### 2.3.2. Daily Caffeine Intake

Participants’ daily caffeine intake was calculated by combining product caffeine content information with the consumption frequency data obtained in the questionnaire.

Consumption frequencies (“never”’ to “6+ times per day”) were given a numerical factor to quantify daily intake; for example, if a product was consumed once per week, the factor was 1/7 = 0.143. Where a frequency range was given, the middle value of the range was used (e.g., for products consumed 4–5 times per day, the factor would be 4.5). Data for 11 participants who reported consuming no caffeine were removed when calculating the median daily caffeine intake.

For each source category, total caffeine intake was calculated by tallying each of the products in that category together (e.g., black tea, green tea, and iced tea consumption was combined for the “Tea” category). Likewise, the estimated total caffeine intake was calculated by aggregating the sum of each caffeine source (i.e., the sum of total intake for each of the following categories: tea, coffee, chocolate, kola drinks, energy drinks, caffeinated RTDs, sports supplements, and caffeine tablets) to give the total daily caffeine intake,; full details including methods of preparation of each source are reported elsewhere [16].

#### 2.3.3. Key Motivators for Caffeine Consumption by Source

Participants who regularly consumed a caffeine source were given a choice of several possible statements about why they might choose to consume this product. Responses were recorded on a Likert scale (options were “strongly agree”, “agree”, “disagree” or “strongly disagree”). Positive responses to a statement were reported as the sum of participants who responded “strongly agree” or “agree”.

#### 2.3.4. Non-Consumption of Caffeinated Products

Participants were provided with a choice of nine potential barriers that might lead to infrequent or non-consumption of caffeinated products. The potential barriers were “there is too much sugar in it”, “it is too expensive”, “it isn’t good for me”, “it has too much caffeine in it”, “I react badly to it”, “I have never considered taking it”, “I don’t want to be dependent on it”, “I don’t like the flavour”, and “I don’t consume caffeine for medical reasons”. The percentage of participants that agreed with each statement for each product was then calculated.

### 2.4. Statistical Analysis

Data was downloaded from the Qualtrics software as Excel files and analysed using SPSS (IBM SPSS Statistics for Windows, Version 25.0. Armonk, NY, USA ).

Variables were tested for normality using the Kolmogorov−Smirnov and Shapiro−Wilk tests and for homogeneity using Levene’s test. Normally distributed data were expressed as mean ± SD, and data not normally distributed were expressed as a median [25th–75th percentiles]. *p* < 0.05 was considered statistically significant. Where variables show statistically significant differences, the effect size was calculated using the following formula: effect size = Z/√n (Mann−Whitney U test). An effect size value of 0.1 indicates a small effect, a value of 0.3 indicates a medium effect, and a value of ≥ 0.5 indicates a large effect [18].

## 3. Results

### 3.1. Participant Characteristics

A total of 216 participants with a mean age of 16.6 years ± 0.82 years (range 15–18 years) completed the questionnaire and their results were analysed. Table 1 outlines participant demographic information (gender, employment status, and BMI).

### 3.2. Consumption of Caffeinated Products by Source

Table 2 shows the number and proportion of participants who consumed caffeine from each source, reported as the total number and proportion of participants, and by gender and employment status. There were 204 participants who consumed caffeine, with a median daily caffeine consumption of 68.3 mg·kg^−1^·day^−1^ [52.3–81.7 mg·kg^−1^·day^−1^].

Girls were 2.25 times more likely than boys to consume tea (*p* = 0.005; Cohens r = 0.165), whereas boys were 2.18 times more likely to consume energy drinks (*p* = 0.01; Cohens r = 0.21) than girls. There was a trend for girls to consume more coffee (*p* = 0.066) and chocolate (*p* = 0.084) than boys. Participants who were in paid employment were 1.93 times more likely to drink coffee than those not in paid employment (*p* = 0.031; Cohens r = 0.168). Those in paid employment were also 3.23 times more likely to drink caffeinated RTDs than those who were not employed (*p* < 0.001; Cohens r = 0.233).

#### 3.2.1. Daily Caffeine Intake

Table 3 shows participants’ daily caffeine intake from all caffeine sources by gender and employment status. Total caffeine intake did not differ between genders, even when adjusted for body weight (*p* > 0.05). The median estimated daily intake of caffeine from tea was higher in girls than in boys (*p* = 0.019). Conversely, the median estimated daily intake of caffeine from kola drinks and energy drinks was higher in boys than girls (*p* = 0.003).

The median estimated daily intake of caffeine from coffee and caffeinated RTDs was higher for adolescents who were in paid employment than those who were not employed (*p* = 0.021 and *p* < 0.001, respectively). The analysis of the type of coffee consumed found a trend toward the increased intake of filter/plunger coffee in employed adolescents (*p* = 0.065) compared to those who were not employed.

#### 3.2.2. Key Motivators for Caffeine Consumption by Source

Table 4 presents the key motivations participants gave for consuming each caffeine source. The taste was the main motivator for the consumption of both tea (90.1%) and chocolate (97.3%). Coffee drinkers reported both taste and energy as the primary motivators for consumption (87.7% and 83.6%, respectively). Experiencing a cold and refreshing drink was the main motivator for kola drink consumption (94.2%), and energy drinks were consumed for their energizing effects (85.0%). Participants consumed sports supplements for energy (61.2%), as a result of peer pressure (61.2%), and as a substitute for illegal drugs (61.2%).

### 3.3. Non-Consumption of Caffeinated Products

Of the total respondents (*n* = 216), 58.8% and 31.3% reported never/infrequently drinking tea and coffee, respectively, due to the flavour. Moreover, 45.3% of participants reported not consuming coffee due to concerns about becoming dependent on it; whereas only 17.0% gave this as a reason for not consuming tea. Participants avoided chocolate, kola drinks, and energy drinks due to health concerns such as high sugar content (61.4%, 56.6%, and 39.0%, respectively). There were 85.4%, 72.0%, 58.3%, and 37.1% of respondents who had never considered taking caffeine tablets, caffeinated sports supplements and gels, caffeinated RTDs, and energy drinks, respectively.

### 3.4. Excess Consumption of Caffeinated Products

There were 21.1% of participants (22.7% of girls and 17.5% of boys; *p* = 0.425) who exceeded the EFSA safe-caffeine intake level of 3 mg·kg^−1^·day^−1^ [13]. Coffee drinkers were 7.86 times more likely to consume caffeine in excess of the safe level than non-coffee drinkers (*p* < 0.001). Tea drinkers were 5.89 times more likely to consume over the safe level than non-tea drinkers (*p* < 0.001). Similarly, energy drink consumers were 2.44 times more likely to consume over the safe caffeine level than non-energy drink consumers *(p* = 0.021). A trend also exists between the consumption of caffeinated RTDs and exceeding the safe level of caffeine intake (*p* = 0.054).

### 3.5. Perceived Effects from Caffeine Consumption

**Figure 1:**
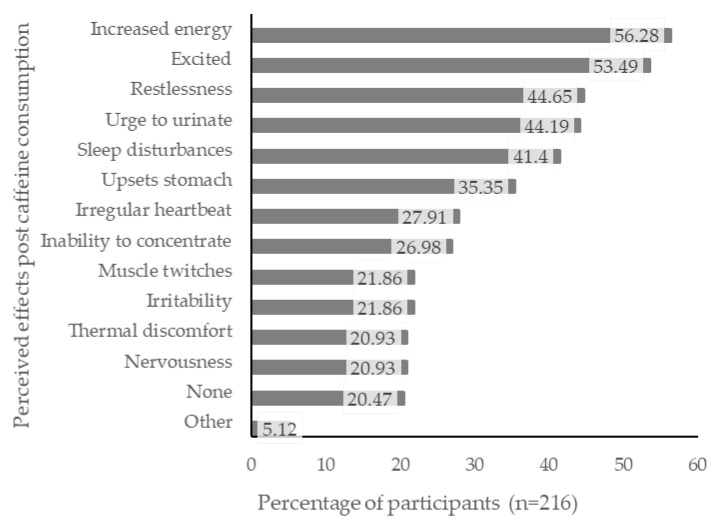
Self-reported “perceived effects” experienced post-caffeine consumption (*n* = 216).

Most participants (79.5%) reported experiencing at least one type of perceived effect following caffeine consumption, the most frequent being “increased energy” (56.3%), “excited” (53.5%), “restlessness” (44.7%), “urge to urinate” (44.2%), and “sleep disturbances” (41.4%; Figure 1).

## 4. Discussion

Our findings indicate that the proportion of adolescents who consume caffeine daily may have risen from 71% in 2008/2009 to 94.9%, as observed in the present study [10]. Chocolate, coffee, kola drinks, and tea were the most common sources of caffeine (85.1%, 56.3%, 55.4%, and 55.3%, respectively). Although chocolate was consumed by most participants, it was not a significant contributor to caffeine intake due to its low caffeine content and infrequent intake. Coffee was the key caffeine contributor, consistent with previous survey findings in New Zealand [10]. In contrast, studies in Canadian, USA, and Croatian adolescents found soft drinks (including kola drinks) were the most frequently consumed caffeinated beverage [9,15,19]. These differences may be due to product accessibility, which is known to influence intake, as soft drinks are frequently available for sale in schools in the USA and Canada [15]. Alternatively, these patterns may reflect cultural norms, with New Zealand having a prominent “café culture”.

The median daily caffeine intake in this study of 68.3 mg·day^−1^ is similar to that of Americans aged 13 to 17 (52 to 66 mg·day^−1^) [20,21] but higher than intakes reported for Australians aged 14 to 16 (41.7 mg·day^−1^) and South Korean adolescents aged 15 to 18 (30.04 mg·day^−1^) [22]. Conversely, a higher mean daily caffeine intake of 149.2 mg·day^−1^ has been reported among 32,000 European adolescents aged 10 to 18 [23], possibly reflecting cultural preferences.

The median caffeine intake in this study (1.21 mg·kg^−1^·day^−1^) is well below the adverse effect level (3 mg·kg^−1^·day^−1^) suggested by EFSA [13]. However, one-fifth (21.1%) of participants did exceed this amount daily; a three-fold increase over the 7% of New Zealand adolescents reported to exceed the safe level in 2008/2009 [10], and greater than the 8% reported among Icelandic 15-year-olds (*n* = 3310) [24] and 12.2% Polish adolescents 16 to 18 years who exceeded this safe level (*n* = 508) [25]. While some of this increase may be due to the inclusion of a wider range of caffeinated products or the emergence of products with a greater caffeine content, it may also represent an increasing group of high-caffeine consumers. It is this group, who regularly exceed the daily safe level of caffeine intake, who would benefit most from education about the benefits and risks of caffeine use, and strategies are needed to improve adolescents’ “caffeine literacy” and negate excess caffeine intake [26].

We found that nearly one-third (31.6%) of adolescents consumed energy drinks, a ten-fold increase over that reported in 2008/2009 (3.1%) [10]. This is likely the result of increased market availability of energy drinks in the intervening decade. Since total caffeine intake has been relatively stable over the past 10 years, adolescents may be replacing more traditional sources of caffeine with energy drinks. Energy drink consumption in New Zealand was lower than among Icelandic 15-year-olds (47%) [24] but higher than among Korean middle and high school students (23.9%) [27]. Energy drink intake has been associated with risky or negative health behaviour, including sensation seeking, poor sleep patterns, smoking, and use of alcohol and illicit drugs [28,29,30]. These behaviours frequently begin during adolescence, but whether energy drink usage and negative behaviour occur among New Zealand adolescents is yet to be determined.

We found boys had greater caffeine intake from energy drinks than girls, which is consistent with previous studies in the USA, Canada, and Poland [9,28,30,31,32]. Boys were 2.18 times more likely to consume energy drinks than girls, which may relate to the way these products are marketed, as studies have found that energy drinks are viewed more as a “drink for males” and advertising is aimed at young men [33,34]. Therefore, efforts to reduce the negative effects of caffeine from energy drinks should focus on advertising and labelling regulations, which target boys [14,34]. Energy drinks are subject to labelling regulations under the New Zealand 2010 Food Standards code, and thus there is potential for them to display warning labels [26].

Caffeine intake from tea was 2.25 times higher in girls than boys, which may relate to the idea that tea is perceived as a more “feminine” drink [9,31]. These findings illustrate that dietary caffeine sources differ between boys and girls and suggest different strategies are needed to target high-caffeine consumers.

Although the current study found no difference in the total caffeine intake between those who are employed and those who are not, employed adolescents were 1.93 times more likely to drink coffee and 3.23 times more likely to drink caffeinated RTDs than those who were not employed, with a trend (*p* = 0.065) towards increased filter/plunger coffee intake in those employed. Again, this trend may be due to improved accessibility of coffee at their place of employment [15], or adolescents may feel the need for more coffee to give them energy to work. Additionally, employed adolescents will likely have greater disposable income than their non-employed counterparts, money they can spend on caffeine containing products if they choose [29].

Taste was identified as a significant motivator promoting the consumption of all caffeinated products except for sports supplements and gels. Tea was also consumed for the attributes of temperature as well as to relax, suggesting tea drinkers view their tea consumption as habitual rather than for the caffeine content. This is consistent with a Serbian study where adolescents identified their main reason for drinking tea “as a habit” [35].

Many participants who did not consume coffee based their decision on concerns about becoming dependent on caffeine (45.3% of non-consumers); however, participants were less concerned about becoming dependent on tea (17.0% of non-consumers gave this as a reason to avoid tea) despite both products providing a significant amount of caffeine (coffee: 83–350 mg/serving; tea 57 mg/serving) [36].

Coffee drinkers and energy drink users identified the energising effects (e.g., “to stay awake”, “for energy” and “to wake up”) as the main reason for consuming these beverages. This is consistent with previous findings from a Saudi Arabian study (*n* = 1006) that adolescent boys ingested coffee “to stay awake” [35] and energy drinks to “boost energy” [35]. A qualitative Canadian study (*n* = 166) found that energy provision was the main motivating factor regardless of whether they liked the taste of caffeinated beverages [15].

Unlike previous studies in NZ and Australia, which identified that peer pressure or “fitting in” was a key factor in energy consumption among those aged 16 to 21, in the present study, peer pressure was not explicitly identified by participants as a major reason for consuming caffeinated products, except for sports supplements [14,34]. However, our participants identified eating or drinking these products “with friends”, or “whenever offered” as major motivators for consuming tea, coffee, chocolate, kola drinks, and caffeinated RTDs; therefore, peer pressure may be a subconscious influence on adolescent caffeine consumption.

Previous studies have identified the effect of advertising as a significant trigger for caffeine consumption, specifically energy drink intake in adolescents [34,37]. However, this was not identified as a major reason for caffeine consumption in the present study. The effects of advertising may occur at a subconscious level, as this age group has a high level of exposure to energy drink advertising [38]. The methods used in the present study may not have been sensitive enough to elucidate the relationship between caffeine and advertisements in New Zealand.

Sports supplement and gel usage in this study were low, with only 2.8% of participants reporting that they use these regularly. These products are generally used by athletes to improve performance as an ergogenic aid [39]. However, when asked about their key motivations for consuming these products, participants reported “peer pressure” and “as a substitute for illicit drugs” in addition to the expected response of “for energy”. This is potentially problematic behaviour and should be investigated further as it may indicate sensation seeking, which has previously been related to energy drink consumption [40].

Most participants (79.5%) reported experiencing at least one type of perceived effect following caffeine consumption, with the most frequently reported being increased energy, excitement, restlessness, the urge to urinate, and sleep disturbances. Previous reports have suggested that adolescents consuming caffeinated products expect to experience increased energy [14,29], altered mood [41], and increased wakefulness [42,43].

The adolescents who reported perceived adverse effects had a higher intake of coffee and energy drinks (*p* < 0.001) than participants who reported no adverse effects following caffeine consumption. Since these participants reported consuming these products for their energizing benefits, they are aware of the caffeine content of these products and expect to experience these effects following consumption. Adolescents have a good understanding of the links between energy and coffee and a good knowledge of the effects of caffeine, including short-term increases in energy, rapid heart rate, jitteriness, and insomnia [15,29]. This suggests that the negative effects of caffeine were not a deterrent to caffeine intake and were unlikely to impact consumption.

This study adds to the body of research about adolescent caffeine intake patterns, and the motivations for caffeine use may help inform public health strategies (education, labelling, advertising regulations) to support healthy caffeine habits among adolescents by increasing their “caffeine literacy” [26].

Although the study has significant strengths, including using CaffCo, a New Zealand-validated questionnaire to determine caffeine intake patterns in New Zealand, there are also limitations. The use of a regional-based convenience sample and the relatively small sample size may have affected the accuracy of the caffeine consumption data and reduced the generalisability of results. Another limitation is that caffeine non-consumers may have felt the questionnaire was not applicable to them and therefore they did not participate, leading to an overestimation of the proportion of adolescent caffeine consumers. Future research should use a larger, more diverse sample, allowing for cluster analysis to better identify caffeine user types and determine how caffeine consumption patterns change over the life course.

## 5. Conclusions

Most New Zealand adolescents regularly consume caffeine, with the majority having a moderate intake. Coffee is the main contributor to daily caffeine intake, followed by chocolate and tea. Both gender and employment status affect the source but not the overall level of caffeine intake. Taste, energy, and temperature were the main reasons adolescents chose to consume caffeinated products, with a small number of adolescents using sports supplements due to peer pressure or instead of illicit drugs. Over the past decade, there has been a ten-fold increase in caffeine intake from energy drinks. A fifth (21.1%) of New Zealand adolescents had a daily caffeine intake above the recognised safe level, which represents a three-fold increase over the past decade. Individuals consuming over the safe, recognised caffeine level had a higher intake of coffee, tea, and energy drinks than those who did not exceed the safe intake level and were also more likely to experience adverse effects following caffeine intake. Adolescents who consume greater than the safe level of caffeine would benefit from strategies designed to improve caffeine literacy to reduce the risk of caffeine-related harm.

## Figures and Tables

**Table 1:** Participant characteristics by gender.

Participant Characteristics	Total, *n* (%)	Boys, *n* (%)	Girls, *n* (%)
Gender ^a^	216 (100)	75 (35.0)	139 (65.0)
Employment status (*n*= 192)			
No paid employment	123 (64.1)	41 (64.1)	77 (63.1)
Paid employment	69 (35.9)	23 (35.9)	45 (36.9)
BMI groups ^b^ (*n*= 133)			
Thin/Underweight	1 (0.75)	0 (0.0)	1 (1.2)
Normal	105 (78.9)	40 (80.0)	64 (78.0)
Overweight	24 (18.0)	9 (18.0)	15 (18.3)
Obese	3 (2.3)	1 (2.0)	2 (2.4)

^a^ Two participants reported their gender as “other”; ^b^ BMI: Body Mass Index WHO BMI-for-age charts for 5–19 years [17].

**Table 2:** Number and proportion of caffeinated products consumed overall and by gender and employment status.

Caffeine Source	All Participants, *n* (%) (*n* = 215) ^a^	Boys, *n* (%) (*n* = 74) ^a^	Girls, *n* (%) (*n* = 139)	Pearson’s Chi-Square Test (ꭕ^2^)	*p*-Value ^b^	Odds Ratio	No Paid Employment, *n* (%) (*n* = 123)	Paid Employment, *n* (%) (*n* = 69)	Pearson’s Chi-Square Test (ꭕ^2^)	*p*-Value ^b^	Odds Ratio
All sources	204 (94.9)	69 (93.2)	133 (95.7)	-	0.520 ^c^	-	119 (96.7)	68 (98.6)	-	0.665 ^c^	-
Tea	119 (55.3)	31 (41.9)	86 (61.9)	7.786	0.005 ^d^	2.25	73 (59.3)	35 (50.7)	1.336	0.248	-
Coffee	121 (56.3)	35 (47.3)	84 (60.4)	3.379	0.066	-	66 (53.7)	48 (69.6)	4.637	0.031 ^d^	1.93
Chocolate	183 (85.1)	59 (79.7)	123 (88.5)	2.980	0.084	-	111 (90.2)	62 (89.9)	0.007	0.931	-
Kola drinks	117 (54.4)	46 (62.2)	70 (50.4)	2.712	0.100	-	73 (59.3)	37 (53.6)	0.592	0.441	-
Energy drinks	68 (31.6)	32 (43.2)	36 (25.9)	6.684	0.010 ^d^	2.18	41 (33.3)	23 (33.3)	0.000	1.000	-
Caffeinated RTD	47 (21.9)	18 (24.3)	28 (20.1)	0.498	0.480	-	19 (15.4)	26 (37.7)		<0.001 ^d^	3.23
Sports supplements	6 (2.8)	3 (4.1)	3 (2.2)	-	0.421 ^c^	-	3 (2.4)	3 (4.3)	-	0.669 ^c^	-
Caffeine tablets ^e^	1 (0.5)	-	-	-	-		-	-	-	-	-
Consumes no caffeine	11 (5.1)	5 (6.8)	6 (4.3)	-	0.520 ^c^	-	4 (3.3)	1 (1.4)	-	1.000 ^c^	-

^a^ One participant was removed from the intake analysis due to excessively high reported caffeine (removed as outlier); ^b^ Pearson’s chi-square test used; ^c^ Fisher’s exact test used (minimum expected count < 5); ^d^ Significant result (*p* < 0.05). ^e^ Only one participant reported consuming caffeine tablets, so this source was removed from subsequent analysis.

**Table 3:** Daily caffeine intake based on source by gender and employment status.

Caffeine Source	All Participants (mg·day^−1^) (*n* = 215) ^a^	Boys (mg·day^−1^) (*n* = 74) ^a^	Girls (mg·day^−1^) (*n* = 139)	Mann−Whitney Test Statistic (U)	*p*-Value ^b^	Effect Size ^®^	No Paid Employment (mg·day^−1^) (*n* = 123)	Paid Employment (mg·day^−1^) (*n* = 69)	Mann−Whitney Test Statistic (U)	*p*-Value ^b^	Effect Size ^®^
All sources	68.3 (4.9, 158.9)	52.3 (18.3, 109)	67.6 (28.7, 167)	3986.0	0.126	-	68.3 (20.4, 134.3)	67.1 (29.2, 194)	3722.0	0.363	-
Tea	3.71 (0.00, 25.2)	0.00 (0.00, 18.3)	4.33 (0.00, 26.5)	3702.0	0.019 ^c^	0.165	4.64 (0.00, 27.0)	0.468 (0.00, 23.2)	3604.5	0.197	-
Coffee	10.7 (0.00, 88.6)	2.04 (0.00, 55.0)	17.4 (0.00, 90.9)	4041.5	0.150	-	5.87 (0.00, 75.4)	31.0 (0.00, 116)	3251.0	0.021 ^c^	0.168
Chocolate	6.28 (2.58, 11.4)	5.87 (2.58, 10.0)	6.72 (2.70, 11.3)	4088.5	0.204	-	7.12 (2.70, 11.7)	5.96 (2.70, 11.8)	3828.5	0.541	-
Kola type drinks	2.25 (0.00, 9.25)	5.53 (0.00, 18.2)	1.06 (0.00, 5.56)	3466.5	0.003 ^c^	0.209	3.62 (0.00, 8.84)	0.863 (0.00, 11.1)	3698.5	0.312	-
Energy drinks	0.00 (0.00, 3.18)	0.00 (0.00, 19.8)	0.00 (0.00, 1.86)	3601.0	0.003 ^c^	0.210	0.00 (0.00, 5.20)	0.00 (0.00, 3.18)	3968.5	0.805	-
Caffeinated RTD	0.00(0.00, 0.00)	0.00(0.00, 0.71)	0.00(0.00, 0.00)	4282.0	0.289	-	0.00(0.00, 0.00)	0.00(0.00, 1.54)	3197.0	0.001 ^c^	0.233

Values are medians [25th, 75th percentile]. ^a^ One participant was removed from the intake analysis due to excessively high reported caffeine (removed as outlier); ^b^ Mann−Whitney test used; ^c^ Significant result (*p* < 0.05).

**Table 4:** Percentage agreement of motivation for caffeinated product consumption ^a^.

Motivators	Tea (%)	Coffee (%)	Chocolate (%)	Kola Drinks (%)	Energy Drinks (%)	Caffeinated RTDs (%)	Sports Supplements (%)
Taste	90.1	87.7	97.3	91.0	72.6	74.0	-
Temperature (Warm ^b^ /cold ^c^)	88.4 ^b^	81.2 ^b^	71.5 ^b^	94.2 ^c^	73.9 ^c^	60.0 ^c^	-
To comfort and relax myself	88.4	52.4	64.5	-	-	26.0	-
Whenever it is offered to me	81.0	73.7	75.8	72.7	64.4	76.0	-
Because it is easily available	79.3	77.1	63.4	64.4	49.3	-	-
To wake up	34.7	87.7	-	-	72.6	-	-
To stay awake	-	85.3	-	-	82.2	-	-
For energy	26.4	83.6	-	37.2	85.0	-	61.2
As a treat	-	-	87.7	82.7	-	-	-
With friends	58.7	75.4	84.4	82.7	63.0	90.0	25.0
For physical energy	-	67.2	-	-	72.6	20	23.1
For the alcohol content	-	-	-	-	-	84.0	-
Others are eating/drinking it	29.7	46.7	47.3	38	26.0	72.0	38.5
Peer pressure	>10	12.3	>10	>10	12.30	22.0	61.2
As a substitute for illegal drugs	-	-	-	-	-	-	61.2

^a^ Key motivators expressed as a percentage of participants who reported “agree” or “strongly agree” for each statement by source; ^b^ Warm beverage temperature was a key motivator for caffeine consumption; ^c^ Cold beverage temperature was a key motivator for consumption. - indicates that participants were not asked this statement for that source as it was not applicable.

## Data Availability

All relevant data are presented in this manuscript.
